# Contribution of the astrocytic tau pathology to synapse loss in progressive supranuclear palsy and corticobasal degeneration

**DOI:** 10.1111/bpa.12914

**Published:** 2020-12-29

**Authors:** Nils Briel, Katrin Pratsch, Sigrun Roeber, Thomas Arzberger, Jochen Herms

**Affiliations:** ^1^ German Center for Neurodegenerative Diseases (DZNE) e.V., Site Munich Munich Germany; ^2^ Center for Neuropathology and Prion Research University Hospital Munich Ludwig–Maximilians‐University Munich Germany; ^3^ Munich Medical Research School Faculty of Medicine Ludwig‐Maximilians‐University Munich Germany; ^4^ Department of Psychiatry and Psychotherapy University Hospital Munich Ludwig‐Maximilians‐University Munich Germany; ^5^ Munich Cluster of Systems Neurology (SyNergy) Ludwig‐Maximilians‐University Munich Germany

**Keywords:** astrocytic plaques, corticobasal degeneration, progressive supranuclear palsy, synapse loss, tauopathy, tufted astrocytes

## Abstract

Primary 4‐repeat tauopathies with frontotemporal lobar degeneration (FTLD) like Progressive Supranuclear Palsy (PSP) or Corticobasal Degeneration (CBD) show diverse cellular pathology in various brain regions. Besides shared characteristics of neuronal and oligodendroglial cytoplasmic inclusions of accumulated hyperphosphorylated tau protein (pTau), astrocytes in PSP and CBD contain pathognomonic pTau aggregates — hence, lending the designation tufted astrocytes (TA) or astrocytic plaques (AP), respectively. pTau toxicity is most commonly assigned to neurons, whereas the implications of astrocytic pTau for maintaining neurotransmission within the tripartite synapse of human brains is not well understood. We performed immunofluorescent synapse labeling and automated puncta quantification in the medial frontal gyrus (MFG) and striatal regions from PSP and CBD postmortem samples to capture morphometric synaptic alterations. This approach indicated general synaptic losses of both, excitatory and inhibitory bipartite synapses in the frontal cortex of PSP cases, whereas in CBD lower synapse densities were only related to astrocytic plaques. In contrast to tufted astrocytes in PSP, affected astrocytes in CBD could not preserve synaptic integrity within their spatial domains, when compared to non‐affected internal astrocytes or astrocytes in healthy controls. These findings suggest a pTau pathology‐associated role of astrocytes in maintaining connections within neuronal circuits, considered as the microscopic substrate of cognitive dysfunction in CBD. By contrasting astrocytic‐synaptic associations in both diseases, we hereby highlight astrocytic pTau as an important subject of prospective research and as a potential cellular target for therapeutic approaches in the primary tauopathies PSP and CBD.

## Graphical abstract

The primary tauopathies progressive supranuclear palsy (PSP) and corticobasal degeneration (CBD) show a diverse pattern of cellular inclusions and neuropathological characteristics. In this article, we show a differentiated analysis of synaptic loss in affected brain regions of pure 4R‐tau PSP and CBD cases. While in CBD frontal cortices synapse loss was mainly associated with the disease‐defining astrocytic plaques, a rather general synaptic depletion was apparent in PSP.

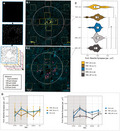

**Abbreviations**
AD: Alzheimer’s disease
ANOVA: analysis of variance
AP: astrocytic plaque
APP: amyloid precursor protein
CA: control astrocyte(s)
CB: coiled bodies
CBD: corticobasal degeneration
Ctrl: control
DF: degrees of freedom
dpi: dots per inch
EAAT2: excitatory amino acid transporter 2
fCtx: frontal cotrex of the middle frontal gyrus
FTD: frontotemporal dementia
FTLD: frontotemporal lobar degeneration
GFAP: glial fibrillary acidic protein
GLT1: glutamate transporter 1
KO: knock‐out
LSM: light sheet microscope
MFG: middle frontal gyrus
NA: numerical aperture
NFT: neurofibrillary tangles
NT: neuropil threads
PBS: phosphate‐buffered saline
PET: positron emission tomography
PSP: Progressive Supranuclear Palsy
pTau: hyperphosphorylated tau
ROI: region of interest
SB: Sudan Black
Str: striatum
SV2A: synaptic vesicle protein 2A
TA: tufted astrocyte
vGAT: vesicular GABA transporter
vGLUT1: vesicular glutamate transporter 1

## 1 INTRODUCTION

The neuropathological classification of frontotemporal lobar degeneration of the tau‐type (FTLD‐tau), a group of neurodegenerative diseases with predominant cognitive (frontotemporal dementia, FTD) and motor impairments, primarily bases on heterogeneous patterns of cytoplasmic inclusions of aggregated hyperphosphorylated *microtubule‐associated protein tau* (pTau) in neurons and glia (9, 16, 18, 27, 48). Differential splicing of exon 10 transcripts of the tau gene leads to 3‐repeat and 4‐repeat tau isoforms. Typical cases of PSP and CBD are associated with a predominant aggregation of 4‐repeat (4R) pTau isoforms (28). In histopathology, the AT8 monoclonal antibody recognizing pTau phosphorylated at both serine 202 and threonine 205 is widely used for visualizing pathological tau deposits (27, 48). Typical CBD cases are characterized by neuronal (pre‐) tangles and ballooned neurons, dense neuropil threads (NT), a prominent white matter pathology with oligodendrocytic coiled bodies (CB) and corona‐like astrocytic plaques (AP), which mainly involve the fronto‐parietal cortices, the striatum as well as the substantia nigra (9, 48).

In contrast, the typical neuropathological traits of PSP are widespread pTau aggregates forming neurofibrillary tangles (NFT), which are sometimes globose, numerous CB, and tufted astrocytes (TA) mainly in the basal ganglia, brainstem, cerebellum and to varying degrees in neocortical areas. The described pathognomonic astrocytic pTau pathology is emphasized in the soma‐distant processes of APs in CBD, whereas in PSP TAs’ inclusions are rather localized in soma‐proximal cell compartments (9, 16, 27, 48).

While higher order pTau assemblies in the form of so‐called “tangles” are thought to have an arguably toxic effect in neurons, lower order pTau oligomers appear to be more potent cellular or synaptic noxae (2, 7, 12, 21, 41). Indeed, recent PET‐imaging studies in human FTD and Alzheimer’s Disease (AD) patients reported (i) a remarkably high synapse loss, (ii) elevated mitochondrial stress marker binding levels, and (iii) a positive correlation between both (preprint: Holland et. al. 2020, medRxiv: 2020.01.24.20018697 and conference report: https://www.alzforum.org/news/conference‐coverage/multimodal‐imaging‐neurodegenerative‐diseases‐links‐pathology‐and‐cellular). Additionally, densitometric approaches with brain lysates obtained from the frontal cortex of AD and PSP subjects showed ca. 50% reductions of synaptophysin protein levels to those of controls, concordant with putatively depleted presynaptic vesicle pools (5, 26). However, a histological validation of a supposed morphological synaptic decrease in FTDs, as suggested by those radio‐ligand or densitometry studies, is lacking to date. Furthermore, whether the synaptic phenotype relates to a cell type‐specific pathology remains unexplored.

Synapse loss is not only a common and characteristic finding in animal models for tau pathology (21, 47, 49), but also a presumptive cause of cognitive deficits in PSP and AD (4, 46). Though, the latter view is challenged by the finding of lower synaptophysin levels in non‐demented vs. demented PSP subjects (5) as well as by more recent findings, which could not confirm decreased binding of the synaptic vesicle protein 2A (SV2A) targeting radio‐ligand [^3^H]UCB‐J to presynaptic vesicle pools in postmortem sections of AD patients in comparison to non‐AD control cases (30).

At the level of cell complexes, the functioning of neuronal circuits in the mammalian brain does not exclusively depend on the cell type‐autonomous physiology of interconnected neurons. There are external factors provided by glial cells that regulate the integrity of neurons and their cellular compartments *in vitro* (25) and *in vivo* (43, 44). The spatial unit an astrocyte is responsible for often is referred to as the “astrocytic domain” or “synaptic island,” when specifying the synaptic responsibility (15, 35). As assessed by comparative studies in humans, non‐human primates, and other species, such domains measure in average about 142 µm in diameter and encompass about 2 million synapses (34, 35). The fine perisynaptic astrocytic processes, being long time presumed as passive bystanders of neuronal communication, emerged as essential components of the tripartite synapse to provide support structurally, trophically, and functionally (36, 38, 43) (preprint: Holt et al. 2019, biorXiv: 10.1101/518787v1). Furthermore, an impaired astroglial support has previously been implicated in a pTau‐ and amyloid precursor protein (APP)‐related disease context, including mouse models recapitulating tauopathies with mutant pTau (P301S, P301L (42), rTg4510 (39)), brain culture internalization approaches (38) and the APP‐KO mouse line (31). In murine hippocampal neuronal‐astrocytic co‐cultures pTau accumulation in astrocytes was followed by diminished gliotransmission and consequent synapse dysfunctions, indicating a direct involvement of astrocytes in the upstream mechanisms of synaptotoxicity (38). Interpreting the neuropathology and astrocytic roles as described before, pTau‐mediated synaptic dysfunction in primary tauopathies is likely to be a joint result of neuronal and astroglial effects.

To address this, we assessed the synaptic density in cortical and striatal areas of PSP and CBD subjects from a morphometry‐centric perspective. We then disentangled cell type‐distinct contributions to the synaptic phenotype and differentiated these effects by the disease context.

## 2 MATERIALS AND METHODS

### 2.1 Human tissue of PSP, CBD and control subjects

#### 2.1.1 Neuropathological evaluation

The neuropathological diagnosis of all cases included was conducted at the Center for Neuropathology, German national reference center for neurodegenerative disorders (23).

At autopsy, the whole brain was dissected out. One hemisphere was frozen immediately. The other one was fixed in formalin for at least two weeks and later cut into 1 cm thick coronal slices. From these, regions of interest including neo‐ and archicortical, basal ganglia, brainstem, cerebellar, spinal areas as well as the hypophysis were cut out, embedded in paraffin and stained for diagnostic evaluation. A board‐examined neuropathologist examined the tissue blocks of all underlying cases. The PSP‐ and CBD cases were classified according to the *NINDS Neuropathologic Diagnostic Criteria* for PSP (16, 27) and the *Office of Rare Diseases Neuropathologic Criteria* for CBD (9).

#### 2.1.2 Selection of cases

4R tauopathy (PSP, CBD) or control cases with significant co‐pathology in areas of interest were excluded from the study. Neurologically and psychiatrically non‐diseased subjects were chosen as control cases. The investigated cohorts were matched for age, postmortem interval (PMI), disease duration, and fixation‐time, and none of these covariates differed significantly between the cohorts (Table 1, Figures S1a–c and S2e). Exclusion criteria for 4R tauopathy (PSP, CBD) cases were immunopositivity for Aβ_42_, TDP‐43, or RD3 (3R tau) in examined regions and lack of pathognomonic cellular pTau inclusion pattern; exclusion criteria for control cases were immunopositivity for Aβ_42_, TDP‐43, AT8, RD3, or RD4 (4R tau) in examined regions. The age atdeath ranged from 52 to 82 years. To address the potential bias of differing fixation durations on the analysis of detected synaptic puncta, studies of correlation showed neither significant relations across all cohorts nor in a cohort‐differentiated view (Figure S2). Thus, the synapse quantification is unlikely biased by this and the other covariates (Figure S1d–k). To be noted, we were limited by the availability of (i) rare formalin‐fixed brain tissue of PSP and CBD cases, in which the astrocytic domain had to be captured within thick vibratome‐sections in its largest diameter and (ii) of those cases with pure tau‐pathology to exclude additional confounding effects by other proteinaceous aggregates.

**TABLE 1:** Covariates of included PSP, CBD and control subjects

Code	Diagnosis	Age (years)	PMI (hr)	Gender	Fixation time (years)	CERAD	BRAAK & BRAAK (NFT)	THAL‐phase (Aβ)	TDP‐43	Disease duration (years)	Locus
103	Ctrl	61	15	Female	9.0	0	1	0	neg	–	MFG/NCau
110	Ctrl	72	23	Male	8.8	0	2	0	neg	–	MFG/Put
111	Ctrl	82	63	Male	7.5	0	1	1^a^	neg	–	MFG/NCau
102	PSP	68	38	Male	3.3	0	1	0	neg	6.0	MFG/NCau
105	PSP	77	78	Female	6.8	0	0	0	neg	2.5	MFG/NCau
107	PSP	64	106	Male	5.2	0	0	0	neg	4.5	MFG/NCau
104	CBD	52	14	Female	7.7	0	0	0	neg	4.5	MFG/NCau
108	CBD	56	44	Male	3.5	0	1	0	neg	2.5	MFG/Put
109	CBD	75	33	Female	5.9	0	0	0	neg	3.0	MFG/NCau

^a^ A*β* plaques were not observed in the frontal cortex.

Abbreviations: A*β*, amyloid beta; Ctrl, control; MFG, medial frontal gyrus; NCau, caudate nucleus; NFT, neurofibrillary tangles; Put, putamen; neg, negative.

#### 2.1.3 Regions of interest

PSP, CBD, and control samples used for this study stem from formalin‐fixed archival brain tissue and corresponding paraffin‐embedded specimen. In coronal brain slices, we sampled circa 1 cm^3^‐measuring tissue blocks from the medial frontal gyrus at the height of the anterior striatum (MFG, Brodmann area: 46) and from the anterior striatum (caudate nucleus at the coronal height of the Ncl. accumbens until the height of the pallidum or from the putamen) of grey and parts of white matter (see Table 1 for information on subjects).

### 2.2 Immunofluorescence staining for synapse analysis

Starting with formalin‐fixed archival coronal brain slices of 1 cm thickness fixed for 3.5 to ca. 9 years, samples containing the regions of interest were cut out and divided into smaller blocks of ca. (1 × 1 × 0.5) cm^3^ volume. Then, these blocks were placed in 2 mL reagent tubes and first subjected to antigen retrieval. For this purpose, tissue blocks were incubated in citrate buffer (10 mM, pH 6) overnight before incubating in fresh medium for 20 minutes at 95°C and subsequent cooling to room temperature. Next, using a Leica VT1000E vibratome, 50 µm‐thick sections were prepared. To avoid batch bias, all samples were processed within one common run for each staining combination. The free‐floating immunofluorescent staining procedure was introduced by permeabilization with 2% Triton X‐100 in 1× PBS (PBST) for 16 hr at 4°C. Unspecific potential binding sites were blocked with 10% (v/v) appropriate serum (donkey, #D9663; goat #G9023; Sigma‐Aldrich, Germany) in 0.3% PBST for 5–6 hr at room temperature. Next, primary antibodies diluted in 5% serum in 0.3% PBST were applied in appropriate, previously experimentally determined concentrations (Table 2) at 4°C on a shaking platform for three consecutive days. After washing, secondary antibodies were applied in a 1:1000 dilution in 5% serum in 0.3% PBST at room temperature for 4 hours before washing. Quenching of mainly lipid‐caused autofluorescence was achieved by an immersion in 0.02% (w/v) Sudan Black (SB) in 70% (v/v) ethanol for 2 minutes. Finally, sections were mounted onto Superfrost^®^‐plus slides (Thermo Fisher Scientific, Germany) and covered with Fluorescence Mounting Medium (#S302380‐2, Agilent Dako, Germany) and #1.5H high‐precision imaging coverslips.

**TABLE 2:** Antibodies and respective usage specifications

Antibodies list	Dilution	Identifier and source
*Primary*		
Anti‐AT8, mouse	1:200	MN1020, Thermo Fisher Scientific, Germany
Anti‐GEPHYRIN, mouse	1:150	147 011, Synaptic Systems Ltd, Germany
Anti‐GFAP, goat	1:150	ab53554, Abcam, Germany
Anti‐GLT1/ EAAT2, guinea pig	1:250	AB1783, Merck Chemicals Ltd, Germany
Anti‐HOMER1, guinea pig	1:110	160 004, Synaptic Systems Ltd, Germany
Anti‐HOMER1, rabbit	1:100	160 002, Synaptic Systems Ltd, Germany
Anti‐vGAT, rabbit	1:200	131 008, Synaptic Systems Ltd, Germany
Anti‐vGLUT1, rabbit	1:100	ZRB2374, Sigma‐Aldrich Chemie Ltd, Germany
*Secondary*		
Anti‐goat, Alexa Fluor®647, donkey	1:1000	A21447, Thermo Fisher Scientific, Germany
Anti‐guinea pig, Alexa Fluor®488, goat	1:1000	A11073, Thermo Fisher Scientific, Germany
Anti‐guinea pig, AlexaFluor®647, goat	1:1000	A21450, Thermo Fisher Scientific, Germany
Anti‐mouse, Alexa Fluor®568, donkey	1:1000	A10037, Thermo Fisher Scientific, Germany
Anti‐mouse, Alexa Fluor®568, goat	1:1000	A11031, Thermo Fisher Scientific, Germany
Anti‐rabbit, Alexa Fluor®647, goat	1:1000	A21244, Thermo Fisher Scientific, Germany
Anti‐rabbit, AlexaFluor®488, donkey	1:1000	A21206, Thermo Fisher Scientific, Germany
Anti‐rabbit, AlexaFluor®488, goat	1:1000	A11008, Thermo Fisher Scientific, Germany

Antibodies used for *excitatory* synapse analysis were rabbit anti‐vGLUT1 and guinea pig anti‐HOMER1 and the fluorescent‐labeled goat anti‐rabbit AlexaFluor^®^647 and goat anti‐guinea pig AlexaFluor^®^488. Mouse anti‐AT8 labeled with goat anti‐mouse AlexaFluor^®^568 was co‐stained to aid orientation, but not used in analysis.

Antibodies used for *inhibitory* synapse analysis were rabbit anti‐vGAT and mouse anti‐GEPHYRIN and the fluorescent‐labeled donkey anti‐rabbit AlexaFluor^®^488 and donkey anti‐goat AlexaFluor^®^647. Goat anti‐GFAP labeled with donkey anti‐goat AlexaFluor^®^647 was co‐stained to aid orientation, but not used for further analysis.

For *astrocytic domain* analyses, we used mouse anti‐AT8, rabbit anti‐HOMER1, guinea pig anti‐GLT1/EAAT2, and the fluorescent‐labeled goat anti‐mouse AlexaFluor^®^568, goat anti‐rabbit AlexaFluor^®^488, and goat anti‐guinea pig AlexaFluor^®^647. See Table 2 for information about antibodies and applied dilutions.

### 2.3 Image acquisition, processing, and synapse analysis

Cover‐slipped tri‐labeled sections were inspected using a Zeiss LSM780 confocal microscopy system (Zeiss, Germany) assisted by the “ZEN black” software and equipped with a Plan Apochromat 40×/NA 1.4 oil DIC M27 objective. Isocortical layers II‐IV or striatal grey matter were identified by their nuclei density or reduced amount of myelinated axon tracts appearing black in SB lipid stain, respectively. Five (50 × 50) µm^2^ large 2‐channel images (pre‐ and postsynaptic) were randomly sampled within the predefined histological area for general synapse quantifications using standardized microscope settings (1024 dpi; 16‐bit, 0.049 µm lateral resolution, pinhole set to 29 (488 nm channel) and 39 µm (647 nm channel). When investigating synapse densities related to astrocytic pathology, 11–14 individual characteristic AT8^+^ astrocytes and 4–6 AT8^‐^/EAAT2^+^ control astrocytes were identified in two representative cases (PSP = 1, CBD = 1, Ctrl = 1) with less pronounced pathology in the cortex of the MFG. The acquisition of a sectioning plane was standardized to the respective astrocyte’s centroid core, recognized as round, “empty” structure in the AT8 or EAAT2 channel (Figure 3b). Then, a (212 × 212) µm^2^‐large 3‐channel image was acquired with standardized settings (HOMER1/AT8/EAAT2, 4096 dpi; 16‐bit, 0.052 µm lateral resolution, pinhole set to 32 (488 nm channel) or 30 µm (568 nm, 647 nm channels).

A custom ImageJ2‐written macro script was used for pre‐processing raw bipartite synapses images, including background subtraction, bandpass filtering, despeckling, sharpening, and thresholding (Figure S3) to account for fixation and staining artifacts. Next, intermediate files were subjected to colocalization and single channel analyses in the “*Synapse Counter*” tool with size parameters adjusted corresponding to developer’s recommendations (https://github.com/SynPuCo/SynapseCounter; accessed 6 Mar 2020).

In contrast, astrocytic domains (plus surrounding area) were binned into 17 (27 × 27) µm^2^‐large ROIs. Aiming at differentiating the synapse density distribution within these domains, we defined 5 Sholl‐like concentric circles represented by center (*n* = 1), close (*n* = 4), mid (*n* = 4), distant (*n* = 4), and out (*n* = 4) bins around each astrocyte’s core. Such circles were referred to as “Sholl‐like area representations.” The “synapse density distribution” was then defined as the consecutive set of “Sholl‐like area representations” from “center” to “out” present in the raw image of one single astrocytic domain (center, close, mid) plus surrounding area (distant, out; Figure 3a,b). Then, a similar pipeline was run on each of these images as described above, with the final outcome measured by “*Analyze Particles…”* in *ImageJ/FIJI*. The custom scripts and a guided analysis workflow are accessible via the public repository GitHub (https://github.com/nes‐b/AstSyns). Single ROI‐values were reorganized into distance circles along area representations, means calculated for each circle in R 3.6.3 and subsequently processed for statistical analysis and graph generation.

### 2.4 Quantification of neuropathological traits

In order to quantify the extent of neuropathological pTau traits such as NFT, TA/AP, and CB, 5 µm‐thick paraffin sections of the MFG were stained by the AT8 antibody (1:200) on a Roche BenchMark Ultra system (CC1 standard program with preboiling). Stained slides were inspected using an Olympus BX50 equipped with a UPlanFI 20× objective (NA 0.50). By randomly sampling 10 visual fields per MFG sample and by manually counting the number of positive cells, total cell counts were reported for respective traits in all fields. For NTs, though, we estimated the extent on a semi‐quantitative scale ranging from 0 = “no thread” to 5 = “dense meshwork.”

### 2.5 Statistics and plots

All statistical tests were calculated in RStudio (version 1.2.5001, R 3.6.3). Shapiro–Wilk testing of normality distribution on single outcome measurements was used to determine downstream group‐wise comparisons of either means (two‐sided parametric *t*‐test) or medians (two‐sided nonparametric Mann–Whitney *U*‐test). For comparisons of more than two groups, pair‐wise testing with Holm–Sidak correction was applied. Bound analyses, e.g. of astrocyte domain synapse density distributions, were done using two‐way ANOVA and Levene‐test of normality confirmation (https://rpubs.com/tmcurley/twowayanova; accessed 6 Mar 2020) followed by the Games‐Howell test for data sets with unequal variance (https://rpubs.com/aaronsc32/games‐howell‐test; accessed 6 Mar 2020). Statistical assessment and graphic illustration in the R environment was mainly supported by the “ggpubr” (https://github.com/kassambara/ggpubr; accessed 10 Mar 2020) and “ggstatsplot” (https://github.com/IndrajeetPatil/ggstatsplot; accessed 10 Mar 2020) packages.

## 3 RESULTS

### 3.1 Excitatory and inhibitory bipartite synapses are reduced in PSP

**FIGURE 1:**
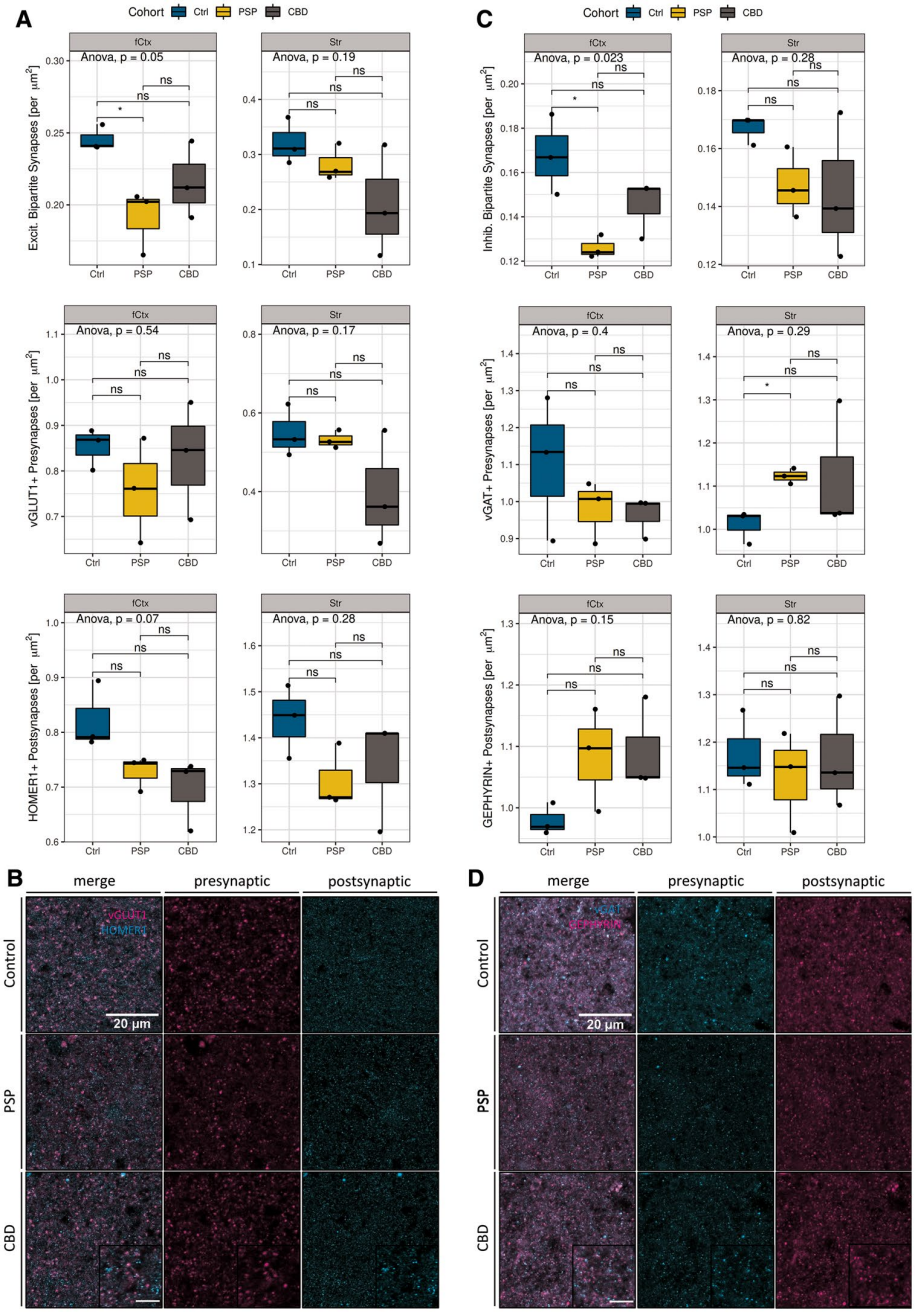
Bipartite synapse quantifications. (a) Statistical analysis of excitatory synapses (# synapses per µm^2^) facetted by region (left column: fCtx, right column: Str) and markers (vGLUT1, HOMER1). Significant reductions of bipartite excitatory synapses in PSP‐fCtx and trending isolated postsynaptic loss in PSP‐ and CBD‐fCtx. Boxplots show synapse densities for colocalized pre‐ and postsynaptic positive (+) signal (1st row), presynapses only (2nd row), and postsynapses (3rd row) in the fCtx and Str. The color code indicates disease entity. Black dots depict values of single cases. The upper and lower hinges of each box correspond to the 75th and 25th percentiles, while median values are represented by a black bar. Whiskers display the range of data within 1.5 of the inter‐quartile range. Significance statements are depicted according to the analysis of variance (ANOVA) with Tukey post hoc correction (entire groups) or *t*‐test (pair‐wise group comparisons). Results are expressed as decimal (ANOVA) or indicated as *p < 0.05 and ns = “not significant” (*t*‐test). (b) Confocal *ex vivo* images of the merged pre‐ and postsynaptic markers for excitatory synapses (vGLUT1 and HOMER1, left), presynaptic (middle column), and postsynaptic (right) in the fCtx of controls (1st row), PSP (2nd row), and CBD (3rd row) subjects. Scale bars: 20 µm (main), 10 µm (inset). (c) Statistical analysis of inhibitory synapses (# synapses per µm^2^) facetted by region (left column: fCtx, right: Str) and markers (vGAT, GEPHYRIN). Significant reductions of inhibitory synapse density in the fCtx and significant increases of inhibitory presynapses in the Str of PSP patients. Depiction and statistical assessment according to (a). (d) Confocal *ex vivo* images of the merged pre‐ and postsynaptic markers for inhibitory synapses (vGAT and GEPHYRIN, left), presynaptic (middle column), and postsynaptic (right) in the fCtx of controls, PSP and CBD subjects. Scale bars according to (c). fCtx, cortex of the MFG; Str, striatum

In order to morphometrically assess alterations in synapse densities, postmortem brain samples from non‐diseased control subjects were compared with those of neuropathologically confirmed PSP and CBD cases with abundant cortical pTau aggregates, but without immunohistochemical signs of cortical or striatal co‐pathology (*n* = 3 per cohort; Table 1). In specimen from cortical tissue from the MFG (fCtx), layer II to IV as well as in grey matter from rostral striatal caudate nucleus or anterior putamen (Str) bipartite synapses were quantified. A bipartite synapse was defined as the unity of colocalized pre‐ and postsynaptic signal to a certain spatial extent (overlap presynaptic channel, postsynaptic channel ≥ 0.33). Here, we used previously established markers for presynapses (excitatory vesicular Glutamate Transporter 1: vGLUT1, inhibitory vesicular GABA Transporter: vGAT; Figure 1b,d) and postsynapses (excitatory HOMER1, inhibitory GEPHYRIN; Figure 1b,d).

Differentiated by synaptic qualities and disease entity, a significant loss of bipartite *excitatory synapses* (vGLUT1+/HOMER1+) could be mapped to the fCtx of PSP patients (Figure 1a 1st row; *t*‐test, *p* = 0.038). No significant alterations were observed in the excitatory bipartite synapse density of the Str in PSP. Noteworthy, while excitatory presynapses remained unchanged, nonsignificant trends became apparent for reduced excitatory postsynapses (HOMER1+) in the PSP‐fCtx (Figure 1, 3, 3rd row; *t*‐test, *p* = 0.099) and PSP‐Str analysis branches (Figure 1, 3, 3rd row; *t*‐test, *p* = 0.099), hinting toward possible latent, isolated excitatory postsynaptic reductions. In the CBD cohort, we did not find any significant synaptic alterations, neither among the anatomical regions of investigation, nor among the separate pre‐, post‐, or bipartite synapse sub‐analyses. However, the excitatory postsynapses’ (HOMER1+) density was trending toward reductions in the CBD‐fCtx analysis branch compared with controls (Figure 1, 3, 3rd row; *t*‐test, *p* = 0.072).

The analysis of bipartite *inhibitory synapses* (vGAT+/GEPHYRIN+) revealed a similar loss pattern regarding colocalization as apparent for excitatory synapses (Figure 1, 3, 1st row; 3 groups one‐way‐ANOVA, *p* = 0.023). In PSP, there were significantly less inhibitory bipartite synapses in the fCtx (Figure 1, 3, 1st row; *t*‐test, *p* = 0.047), but not in the Str. Regarding the separated analyses of single synaptic densities there were almost no significant differences between the PSP and the control group. Interestingly, these counts indicated a significant increase of vGAT+ presynapses in the Str of PSP patients (Figure 1, 3, 2nd row; *t*‐test, *p* = 0.022) — a severely affected brain region in this tauopathy. In the CBD cohort though, neither significant nor trending differences of bipartite or single inhibitory synapse densities were detectable when compared to the levels of the control cohort. Together, in PSP excitatory and inhibitory synapses were reduced in the fCtx, while in the Str only inhibitory presynapes were significantly increased. The assessment of synapses in CBD yielded no significant differences; neither in the fCtx nor in the Str. Nevertheless, there was a trend toward reduced excitatory postsynapses in the fCtx in both PSP and CBD (see Table S1). However, the consistent high synapse density scores of CBD case #109 impelled to ask for a more differentiated questioning toward variable neuropathological features in this disease. Due to clearer results regarding synapse alterations in the cortical than in the striatal regions in these subjects, we focused on the fCtx in the following analyses.

### 3.2 Astrocytic plaques are indicators of a reduced excitatory synapse density in CBD

Since proteinaceous aggregates like pTau assemblies are known for their cell‐harming properties (12, 13), general synaptic alterations, as often observed in neurodegenerative disease (21, 46), would expectedly be linked to the number of cells with pTau aggregates within a certain anatomical region. Thus, we hypothesized, the synaptic density might negatively correlate with the extent of cellular pTau pathology.

**FIGURE 2:**
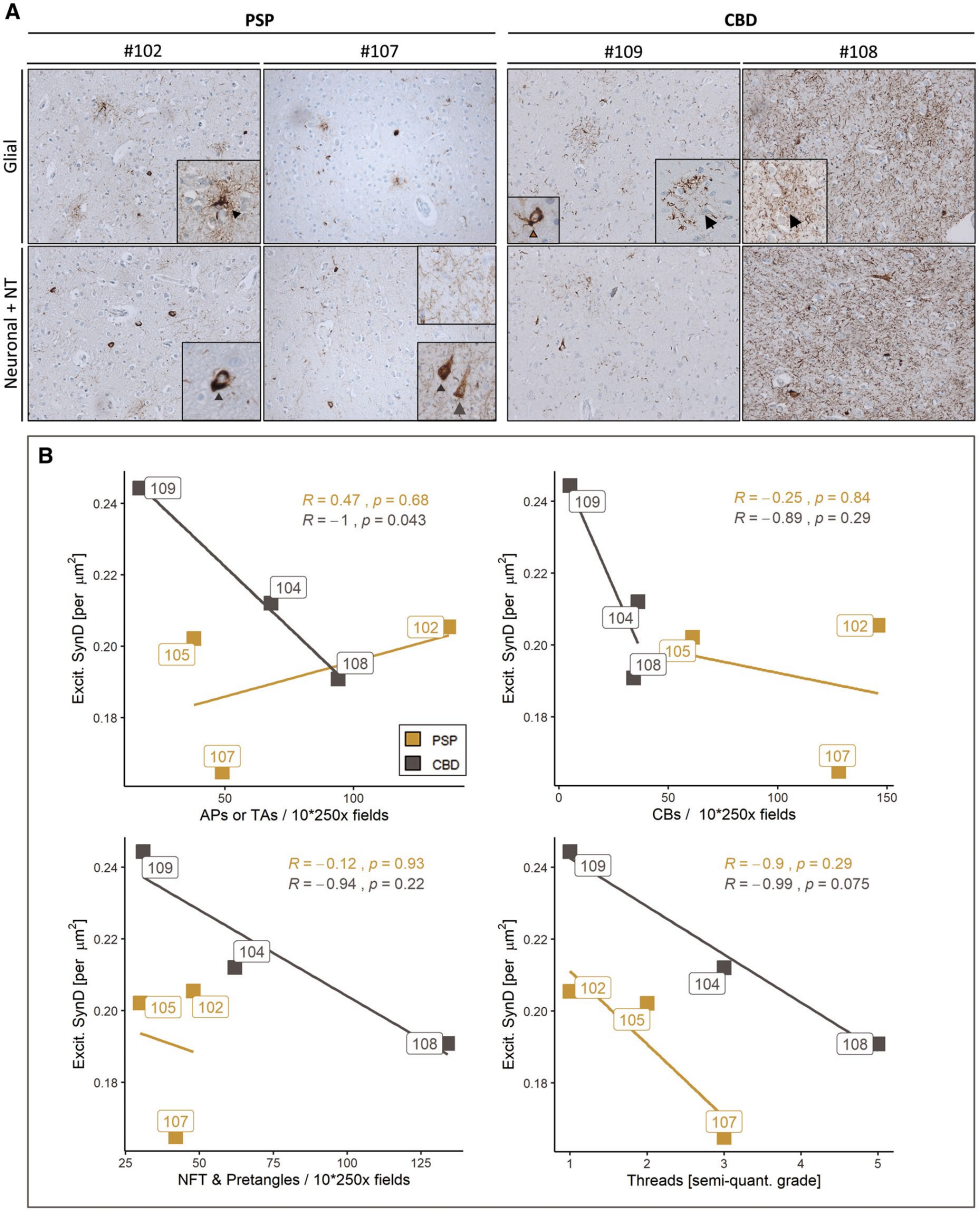
Synapse densities correlate with the occurrence of APs but not TAs in the frontal cortex. (a) Diverse AT8 inclusion pathology in fCtx of investigated PSP and CBD cases visualized by immunohistochemistry using the AT8 antibody. Representative light microscopy images depicting the extent of AT8+ cell type‐assigned neuropathology in those two PSP cases (left panel) and those two CBD cases (right panel) with the highest synapse counts (left column of each panel) and lowest synapse counts (right column of each panel). Insets depict particular AT8 traits of affected brain cell types. The upper row shows glial pathology with TA (arrowhead), APs (arrow), and a CB (brown arrow); the lower row depicts neuronal pathology including pretangles (grey arrowhead), NFTs (grey arrow), together with NT of varying degrees. (b) In the fCtx the density of synapses correlates with neuropathological traits present in CBD (APs, trending with NT/threads), but not with the assessed traits seen in PSP (TAs, NFT, CB, NT/threads). Correlation scatter plots for excitatory synapse density (“Excit. SynD,” synapses per µm^2^ area) in the fCtx facetted by each of the assessed neuropathological traits: TAs and APs (upper left), CBs (upper right), NFTs and pretangles (lower left) as well as NTs (lower right). Color code indicates disease entity. Boxed labels show single case identifiers. Statistical results are expressed as Pearson’s *R* and respective decimal *p* values (see also Table 2). AP, astrocytic plaque; TA, tufted astrocyte

In order to proof this hypothesis, AT8‐labeled paraffin sections adjacent to those samples investigated for synapse counting (MFG, fCtx) were used for obtaining total numbers of cells with different types of pTau aggregates (NFT, TA/AP, CB) and for a semi‐quantitative assessment of pTau positive NT. These values correspond to the observations from 10 randomly sampled fields of 250x magnification (Table 3). Strikingly, when inspecting AT8+ cell‐specific pTau traits of individual CBD cases, #109 had only very few cortical APs, several NFTs and CBs and only few NT in comparison to #108 (Figure 2a). When correlating a given pTau trait with the synaptic density, the extent of astrocytic pTau pathology was the only significant one (Figure 2b, grey; R_PEARSON_ = −1, *p* = 0.043) to estimate excitatory synapse density in CBD, while NT grading was close to significance (Figure 2b, grey; R_PEARSON_ = −0.99, *p* = 0.075). Contrarily, in the PSP cohort none of the pTau aggregate types correlated with synapse reductions (Figure 2b, yellow). In summary, structural synaptotrophic degradation was linked with pTau+ astrocytes in CBD and possibly with neuropil thread pathology. In the investigated PSP cohort though, the factual synaptic reduction was not linked to a singular neuropathological pTau trait.

**TABLE 3:** Quantification of neuropathological traits

Case	Diagnosis	TA/AP	NFT/Pretangles	CB	Threads
102	PSP	137	48	146	1
105	PSP	38	30	61	2
107	PSP	49	42	128	3
104	CBD	68	62	36	3
108	CBD	94	134	34	5
109	CBD	17	31	5	1

Note

Total counts of neuropathological traits per 10 visual 250×‐magnification fields or threads grading in the PSP and CBD fCtx.

Abbreviations: Abbreviations: AP, astrocytic plaque; CB, coiled bodies; NFT, neurofibrillary tangle; TA, tufted astrocytes.

### 3.3 Synapse loss is evident within spatial domains of pTau‐affected astrocytes

**FIGURE 3:**
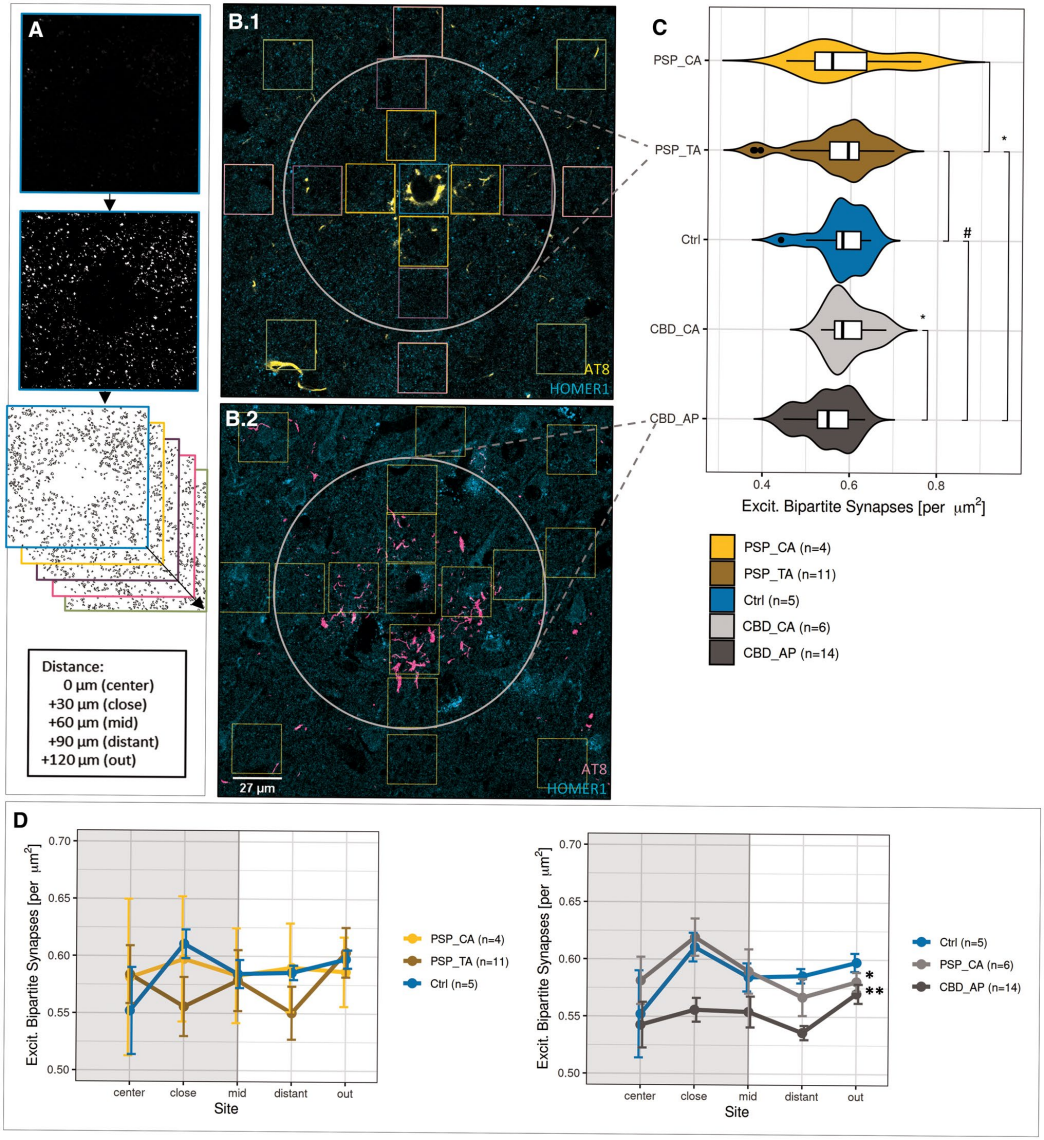
Synapse loss is associated with the territory of APs. (a) Workflow for evaluating astrocytic domain‐associated synapse densities. Bins/ROIs (colored boxes in b) are placed at Sholl‐like, concentric circles surrounding the astrocyte’s core, while somatic targets of the HOMER1 antibody are excluded. Once extracted from the raw image, all bins belonging to one of the five distance representations from “center” to “out” were individually processed and subjected to puncta detection. Merged values of bins belonging to the same distance representation were positioned accordingly and the resulting sequence defined as “synapse distribution.” (b) Exemplifying the image source for the analysis of domain‐associated synapse density AT8 and HOMER1 in TAs in PSP (b.1) and APs in CBD cortices (b.2), where squares delineate bins to extract synapses from. The white circle delimits the astrocytic domain by a priori knowledge. Assignments were given as follows: “center” = light blue, “close” = orange, “mid” = purple, “distant” = pink, “out” = light green. (c) Reduced synapse density in the territory of APs. Combined box‐violin plots depicting the synapse densities of only those bins, which were located within the ascribed astrocytic domain (white circle in b). Comparisons between TAs (golden yellow) / APs (dark grey) and internal AT8‐ control astrocytes (yellow, light grey) of the same condition or external AT8‐ controls (blue) of non‐diseased control subjects. Boxplot description follows Figure 1a. *T*‐test assuming normal distribution, where #: *p* < 0.075, *p < 0.05 and ns: “not significant”. (d) Inherent differences among synapse density distributions within the domains of APs. Means of synapse densities are plotted against area representation assignment for TA and CA in PSP, AP, and CA in CBD and astrocytes in corresponding control cases (left: PSP, right: CBD). The extent of the presumed astrocytic domain is delimited as grey, boxed background. Results are expressed as ±*SEM*, *p < 0.05, **p < 0.01, two‐way‐ANOVA with Leven’s testing for normality and Games‐Howell post hoc test. CA, control astrocytes

In review with the previously assigned reductions in general bipartite synapse counts of both synapse types, we wondered, whether this effect can be ascribed to the single‐cell level. Therefore, we quantified postsynaptic puncta within and surrounding the astrocytic domain, the spatial unit an astrocyte is responsible for 11–14 AT8+ astrocytes as well as 4–6 control astrocytes expressing a marker of neurotransmitter clearance (excitatory amino acid transporter 2, EAAT2) residing in fCtx layers II to VI were identified in 50 µm‐thick sections and imaged. Within this approach synapse densities were determined as HOMER1+ puncta in 17 (27 × 27) µm^2^‐large squared bins placed at concentric circles around each astrocyte’s core (Figure 3a,b). Thereby a total area of ca. 12,400 µm^2^ was covered, the actual astrocytic domain accounting for 6600 µm^2^ (corresponding to 9 squared bins) thereof.

When comparing only *domain*‐assigned bin‐means of synapse densities of pTau+ and pTau‐ astrocytes, APs showed significantly lower values than their internal control astrocytes (CBD_AP vs. CBD_CA, Figure 3c; *t*‐test, *p* = 0.014). In comparison to EAAT2+ astrocytes from non‐diseased subjects, AP domains exhibited at least a trend to sparser synapses (Figure 3c; *t*‐test, *p* = 0.054), whereas examined TA domains did not show such reductions. Instead, TA domains seemed to be less vulnerable to their pTau inclusions, when compared to APs (Figure 3c; *t*‐test, *p* = 0.044).

**FIGURE 4:**
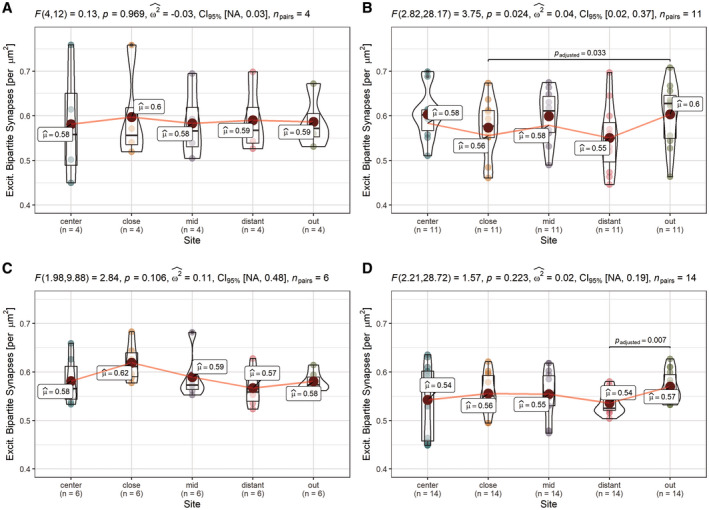
Altered synapse density distributions in domains of APs and TAs. Distribution analysis of single astrocyte cohorts shows unchanged distributions in domains of internal control astrocytes (PSP_CA/CBD_CA donors, AT8‐) and significant differences in the overall distribution in CBD_APs, respective significant between‐bin‐differences to the outermost part around PSP_TAs and CBD_APs approximating a normal synapse density. (a–d) Pair‐wise comparisons of synaptic density among predefined sites within cohorts of astrocyte classes in the fCtx of PSP and CBD subjects. PSP_CA (a), PSP_TA (b), CBD_CA (c), and CBD_AP (d). Graphs show combined box‐violin plots. Small colored dots represent values of single domains at this site, while larger colored dots depict the calculated mean (mean values indicated as boxed labels). Boxed labels provide information on the mean (*µ*). Assuming a normal distribution, Fisher’s repeated measures one‐way ANOVA was used to estimate *F*‐values, *p* values, to determine the effect size (*ω* ^2^) and range of the confidence interval (CI_95%_) given a certain samples size (*n*), as indicated in the caption of each frame. *T*‐testing with Holm–Sidak adjustment was applied for pairwise comparison. Adjusted *p* values of between‐bin‐comparisons are specified as decimals within each of the graphs

To elaborate a potential pTau+ astrocyte‐related synapse depletion as a function of distance, mean densities of all five distances (“center” = 0 µm, “close” = 30 µm, “mid” = 60 µm, “distant” = 90 µm, and “out” = 120 µm) were determined in an ordered fashion, resulting in bound center‐to‐out Sholl‐like area representations of the astrocytic domain (Figure 3a,b). We found first, a consistent initial increment of synaptic densities in the soma‐proximal distance “close” with a subsequent decrease, which was unique to pTau‐ astrocytes (Figures 3d and 4a,c; distance: “close”). Second, the highest mean loss could be assigned to this first distance in TAs in PSP (Figure 4b; “close” vs. “out”; *t*‐test, *p* = 0.033), while the lowest density was measured in the fourth distance of APs in CBD (Figure 4d; “distant” vs. “out”; *t*‐test, *p* = 0.007), which might correspond to an enlarged astrocytic territory size as determined by Oberheim et al. (34) (Figure 3b.2) or to functional consequences extending beyond this arbitrary boundary. Third, these significant differences in spatial synapse distributions levelled out when reaching the last distance (“out”) for TAs in PSP (Figure 4b, two‐way‐ANOVA, *p* = 0.024). Fourth, spatial synapse distributions of APs in CBD were inherently different from those of internal control astrocytes (Figure 3d, Table 4; two‐way‐ANOVA, *p* = 0.003) as well as external control astrocytes (Figure 3d, Table 4; two‐way‐ANOVA, *p* = 0.017). In summary, in this domain‐centered analysis single APs displayed an abnormal synapse distribution at principally reduced density levels, while TAs exhibited only minor declines within the most proximal part of their synaptic islands.

**TABLE 4:** Results of astrocytic domain analysis

Groups	Mean difference	Standard error	*T*‐value	d.f.	*p* value	Upper limit	Lower limit
PSP_CA: PSP_TA	−0.013	0.016	0.593	31.836	0.975	0.051	−0.077
PSP_CA: Ctrl	−0.002	0.015	0.072	26.789	1.000	0.061	−0.064
PSP_TA: CBD_AP	−0.023	0.009	1.845	82.311	0.356	0.012	−0.057
PSP_TA: Ctrl	0.012	0.001	0.829	75.278	0.921	0.051	−0.028
CBD_CA : CBD_AP	−0.036	0.007	3.763	61.596	0.003**	−0.009	−0.063
CBD_CA: Ctrl	−0.002	0.008	0.131	50.205	1.000	0.032	−0.035
CBD_AP: Ctrl	0.034	0.007	3.273	45.153	0.017*	0.064	0.005

Note

Two‐way‐ANOVA with Games‐Howell post hoc correction.

* *p* < 0.05;

** *p* < 0.01.

Abbreviations: AP, astrocytic plaque; CA, control astrocyte; Ctrl, control; d.f., degrees of freedom; TA, tufted astrocyte.

## 4 DISCUSSION

Neurodegeneration in tauopathies has been widely researched in both mouse models and human disease (1, 19, 49). Astrocytes, with specialized responsibilities for structural and functional support within spatially divided territories, modulate neuronal signaling via gliotransmission at the tripartite synapse (3, 22, 32) — an association of pre‐ and postsynaptic neuronal terminals and astrocytic perisynaptic processes (14, 15, 17). Evidently, this led to assumptions of whether and how neuronal circuits depend on the intact function of astrocytes and their peripheral cellular compartments in neurodegenerative disease. Especially, those entities comprising prominent astrocytic inclusion pathology, such as PSP or CBD, attract interest to address these questions.

As synaptic reductions in tauopathies are incompletely characterized (30) (preprint: Holland et al. 2020, medRxiv: 2020.01.24.20018697), we examined synapse alterations in PSP and CBD brains in this study. In addition to morphometric assessments of synapse densities in the frontal cortex and striatal regions of deceased individuals, we integrated the extent of neuropathological traits to elaborate cell type‐differentiated contributions. Further, we found a spatial dependency of synapse densities from pTau+ astrocytes. Thereby, for the first time, our work stresses the pivotal role of astrocytes in maintaining tripartite synapse stability in CBD and confirms a substantial synapse loss in PSP with only minor associations with astrocytic, neuronal, or oligodendroglial pathology.

In general, cognitive decline or motor symptoms might be attributed to (i) synaptic dysfunction occurring primarily on a sub‐synaptic level with only minor morphological synaptic degradation or (ii) to co‐occurring synaptic dysfunction and structural depletion (5, 26, 46). Our findings support the last‐mentioned scenario for both PSP and CBD, as we observed reduced general, non‐trait‐associated synapse reductions in the PSP cohort, while CBD cases exhibited such losses only in correlation with AP pathology or trending with NT. To our knowledge, any differentiation of synaptic losses along with the human pathological astrocytic phenotype, as observed here, has not been shown yet. On the one hand, underestimating the actual effect in territories of PSP‐typical TAs might be due to asymmetrical configuration and distribution of pTau accumulations within the astrocytic domain. On the other hand, this follows a biological notion, in which peripheral pTau deposits in CBD hinder AP astrocytes to sustain intracellular transport to their perivascular endfeet or perisynaptic processes, consequently impairing their neurosupportive functions. On the contrary, TAs being loaded with pTau aggregates more proximally, show only declines in synapse density in this soma‐near part and rather normal levels in the remaining parts of their domains. This could be explained by differences in the distribution of transmembrane transporters or ion channels important for establishing microdomains (e.g. Ca^2+^ channels) along the astrocytic branches (31) and which might allow for compensating compartmentalized dysfunction to different extents between TAs and AP‐astrocytes. In a pathogenetic model shared by TAs and APs, astrocytic tau uptake mechanisms comparable with those involving other potentially neurotoxic compounds to ensure extracellular milieu regulation could take place. Such have been postulated for different tau‐species in a heparin‐sulfate‐dependent manner (29, 40) or in independent, rather unspecified mechanisms in the case of monomeric tau (37). Consequently, in an early phase the AP‐ or TA‐in‐progress might accumulate extracellular tau via suggested import molecules, deposit it as a less toxic aggregated form similar to NFTs and only at a later stage develop dysfunctional synapse support (2, 7, 11, 41).

Hence, another critical component of understanding pTau aggregates and their pathophysiological implications is the discrimination of several tau‐species of hierarchical order (regarding their quartiary structures), phosphorylation patterns and other posttranslational modifications, which are thought to govern disease characteristics (6, 8, 10, 11, 12, 24, 45). To date, determined toxicity is less assigned to higher molecular aggregates such as sarkosyl‐insoluble tau tangles (~1000 monomers) or filaments than rather to truncated, sarkosyl‐soluble forms like oligomeric (~10–100) tau assemblies, which might precede in early tauopathy disease stages (2, 6, 24).

Since a toxic potential of pTau seems to be more evident in relation to APs than to TAs in our study, affected astrocytic subpopulations might be differentially vulnerable to intracellular pTau deposits. Alternatively, disease‐determining cell‐harming properties of astrocytic PSP‐ or CBD‐pTau might underlie this observation. Indeed, PHF‐seeding experiments with PSP and CBD brain extracts in wild‐type mice showed strain‐inherent characteristics in pTau propagation and cellular distribution, further suggesting a diagnostic and etiological separation of these tauopathies is appropriate and necessary (33).

Interestingly, as assessed in the first experiment general synaptic alterations in the CBD cohort were not statistically significant. Given the range of pTau+ cell load in the samples of this cohort in the subsequent correlation analysis, the pathology spread in #109 may not have progressed far enough to reveal a complete region‐assigned synapse loss as detectable by the general synapse density analysis. Nevertheless, a decline was already evident in the synaptic islands of APs in this case, potentially indicating a stage of beginning synaptotoxicity associated with astrocytic pTau inclusions in CBD.

In respect of a vulnerability of synapses differentiated by their excitatory or inhibitory quality, described alterations in PSP argue against gliotransmission‐determined favoring of either one of them. Thus, we assume similar mechanisms to take action in tau‐mediated synaptic deprivation in excitatory as in inhibitory synapses in this disease. Besides this, we did not observe major synaptic derangements in the striatal regions, although inhibitory presynapses were more frequent in this region in PSP brains compared with controls, suggestive of a potential compensation of synaptic dysfunction.

However, it should be noted, that we primarily focused on cases with abundant pTau pathology in the frontal cortex — a rather rare condition in PSP — and without co‐pathology (20). We relied on the availability of archival, non‐embedded brain tissue for free‐floating immunofluorescent staining to allow capturing a sufficient amount of synapses and astrocytic domains in thicker (50 µm) sections. Given the marked synapse loss evident in PET‐ as well as biochemical studies of brains from FTD patients (5) (preprint: Holland et al. 2020, medRxiv: 2020.01.24.20018697), we expected a considerable effect size for synaptic alterations. Therefore, our analysis included only a selected subset of PSP and CBD cases.

In review, this study sets out cellular contributors to synaptic loss in the primary 4R‐tauopathies PSP and CBD, suggesting astrocyte‐mediated synapse loss and the overall pTau pathology as an attribute for general synapse reductions in PSP. Therefore, this study identifies a potential cellular therapeutic target in CBD and emphasizes the usefulness of differentiated pathogenetic and diagnostic considerations regarding these tauopathies. For complementing, our current understanding of the pathogenesis of these diseases, follow‐up studies are needed to validate the neuropathological traits as predictors of synaptic, i.e. factual cognitive impairments in suitable disease models and in larger cohorts of human individuals.

## 5 CONCLUSIONS

Astrocytes as mediators of synaptic transmission and as indicators of pTau inclusion pathology were investigated in the context of the 4R‐tauopathies PSP and CBD. Here, we present evidence for synapse loss associated with APs, the neuropathological hallmark of CBD. In PSP the effects of TA pTau to indicate synapse loss remain behind the impact of the overall pathology. These results implicate pTau‐affected astroglia as contributors to the pathophysiology of synapse loss rather in CBD than in PSP, which is suggestive of cognitive dysfunction in affected patients.

## ETHICS APPROVAL

Collection and distribution of human brain tissue obtained from the Neurobiobank Munich (NBM) respected the principles of informed consent, along with the Code of Conduct established by the BrainNet Europe (23) and were in accordance with the guidelines of the Ethics Committee of the LMU Munich (registration code: 345‐13) and with the 1964 Helsinki Declaration and its later amendments or comparable ethical standards. Prior to death, all individuals donating to the NBM had agreed for autopsy and usage of brain samples in the interest of biomedical research. All cases were double‐pseudonymized in order to account for personal privacy. All experiments of this study were approved by this committee (registration code: 19‐442 KB).

## CONFLICT OF INTEREST

The authors declare that they have no competing interests.

## AUTHOR CONTRIBUTIONS

All authors contributed to the study conception and design. Material preparation, data collection and analysis were performed by NB and KP. The first draft of the manuscript was written by NB and all authors commented on previous versions of the manuscript. All authors read and approved the final manuscript.

## Supporting information

**FIGURE S1** Comparison of subject covariates. (a–c) Pair‐wise comparisons of control, PSP and CBD cohorts regarding disease duration (a), age at death in years (b) and post mortem interval (PMI) (c) of samples used for synapse analysis show no significant differences between cohorts. The upper and lower hinges of each box correspond to the 75th and 25th percentiles, while median values are represented by the black bar. Whiskers display the range of data within 1.5 of the inter‐quartile range, correspondent to the full range of distribution of all 3 cases in the cohort. One‐way‐ANOVA and Welch‐test were used to evaluate group differences, where “ns” >0.05 (d–e) Correlation matrices indicate Pearson’s R (shade) and *p* value >0.05 (crosses) for pairwise correlation tests between covariate data and synapse densities in an cohort‐undifferentiated view for synapse counts in the fCtx. The order of features is given by hierarchical clustering results. Regarding the excitatory synapse analysis, only significant positive correlations between the density of bipartite synapses and pre‐ or postsynaptic densities are observed (d). Regarding the inhibitory synapse analysis, no significant correlations but a negatively correlated pre and postsynaptic density counts are apparent (e). (f–k) The densities of excitatory and inhibitory bipartite synapses in the fCtx are not significantly correlated with the covariates data in a cohort‐differentiated view; neither with disease duration (f,i), pmi (g,j) nor age at death (h,k). Scatter plots of excitatory (f–h) or inhibitory synapse density (g–i) in the fCtx and given covariate. Color code indicates cohort assignment. Boxed labels show single case identifiers. Statistical results are expressed as Pearson’s R and respective decimal *p* values (see also Table 1). Bipart., bipartite; Dur., duration; Exc., excitatory; Inh., inhibitory; SynD, synapse density
**FIGURE S2** Influence of fixation time. (a–b) Representative confocal images of the excitatory bipartite synapse immunofluorescent staining (HOMER1 (a), vGLUT1 (b)) in the fCtx of control, PSP and CBD samples. For each excitatory synapse marker those samples are shown, which were fixed the shortest (respective left column) or the longest (respective right column) within the respective cohort given in rows. Text insets show case identifiers and fixation time in years [y]. (c–d) Scatter plots of the density of excitatory (c) or inhibitory (d) bipartite synapses in the fCtx and the time of formalin‐fixation show no significant correlations. Color code indicates cohort assignment. Boxed labels show single case identifiers. Statistical results are expressed as Pearson’s R and respective decimal *p* values. (e) Pair‐wise comparisons of control, PSP and CBD cohorts regarding formalin‐fixation time of samples used for synapse analysis show no significant differences between cohorts. The upper and lower hinges of each box correspond to the 75th and 25th percentiles, while median values are represented by the black bar. Whiskers display the range of data within 1.5 of the inter‐quartile range, correspondent to the full range of distribution of all 3 cases in the cohort. One‐way‐ANOVA and Welch‐test were used to evaluate group differences. (f) Barplot of single case fixation times. The time in years ranges from 3.3 to 9 years. Exc., excitatory; Inh., inhibitory; SynD, synapse density
**FIGURE S3** Image pre‐processing and synapse quantification workflow. The bipartite synapse densities were quantified from confocal imaging data followed by a consecutive digital image (pre‐)processing workflow. Starting with the light‐sheet confocal imaging of a randomly selected area of 50 × 50 μm^2^ (a) the raw image was preprocessed in Fiji/ImageJ by subtracting background noise, bandpass filtering, despeckling and sharpening (b), as well as thresholding (Yen algorithm) for image binarization. Then the *SynapseCounter* tool (d) was deployed to detect and count single puncta of synaptic markers (e) or to detect pre‐ and postsynaptic signal colocalization (f) in order to quantify corresponding bipartite synapses (g)
**TABLE S1** Quantification of bipartite synapse density. Mean values [per μm^2^] and *p* values of excitatory and inhibitory synapses density analysis in the cortex of the middle frontal gyrus (fCtx, upper part) and in the striatum (Str, lower part) in PSP, CBD and control brains. Exc., excitatory; Inh., inhibitory

## Data Availability

Scripts for pre‐processing and quantifying synaptic puncta images are available on GitHub (https://github.com/nes‐b/AstSyns). Raw data that support the findings of this study are available from the corresponding author upon request.
