# Supplementary material for: Contribution of the astrocytic tau pathology to synapse loss in progressive supranuclear palsy and corticobasal degeneration
Source: Brain Pathol. 2020 Dec 29;31(4):e12914. doi: 10.1111/bpa.12914 (PMC8412068; doi:10.1111/bpa.12914)
Supplement: Supplementary file 1 — FIGURE S1 Comparison of subject covariates. (a–c) Pair‐wise comparisons of control, PSP and CBD cohorts regarding disease duration (a), age at death in years (b) and post mortem interval (PMI) (c) of samples used for synapse analysis show no significant differences between cohorts. The upper and lower hinges of each box correspond to the 75th and 25th percentiles, while median values are represented by the black bar. Whiskers display the range of data within 1.5 of the inter‐quartile range, correspondent to the full range of distribution of all 3 cases in the cohort. One‐way‐ANOVA and Welch‐test were used to evaluate group differences, where “ns” >0.05 (d–e) Correlation matrices indicate Pearson’s R (shade) and p value >0.05 (crosses) for pairwise correlation tests between covariate data and synapse densities in an cohort‐undifferentiated view for synapse counts in the fCtx. The order of features is given by hierarchical clustering results. Regarding the excitatory synapse analysis, only significant positive correlations between the density of bipartite synapses and pre‐ or postsynaptic densities are observed (d). Regarding the inhibitory synapse analysis, no significant correlations but a negatively correlated pre and postsynaptic density counts are apparent (e). (f–k) The densities of excitatory and inhibitory bipartite synapses in the fCtx are not significantly correlated with the covariates data in a cohort‐differentiated view; neither with disease duration (f,i), pmi (g,j) nor age at death (h,k). Scatter plots of excitatory (f–h) or inhibitory synapse density (g–i) in the fCtx and given covariate. Color code indicates cohort assignment. Boxed labels show single case identifiers. Statistical results are expressed as Pearson’s R and respective decimal p values (see also Table 1). Bipart., bipartite; Dur., duration; Exc., excitatory; Inh., inhibitory; SynD, synapse density FIGURE S2 Influence of fixation time. (a–b) Representative confocal images of the excitatory [file BPA-31-e12914-s001.pdf]

Contribution of the Astrocytic Tau Pathology to Synapse Loss in Progressive Supranuclear Palsy and Corticobasal Degeneration

Nils Briel, Katrin Pratsch, Sigrun Roeber,
Thomas Arzberger, Jochen Herms

– Supplemental Data –

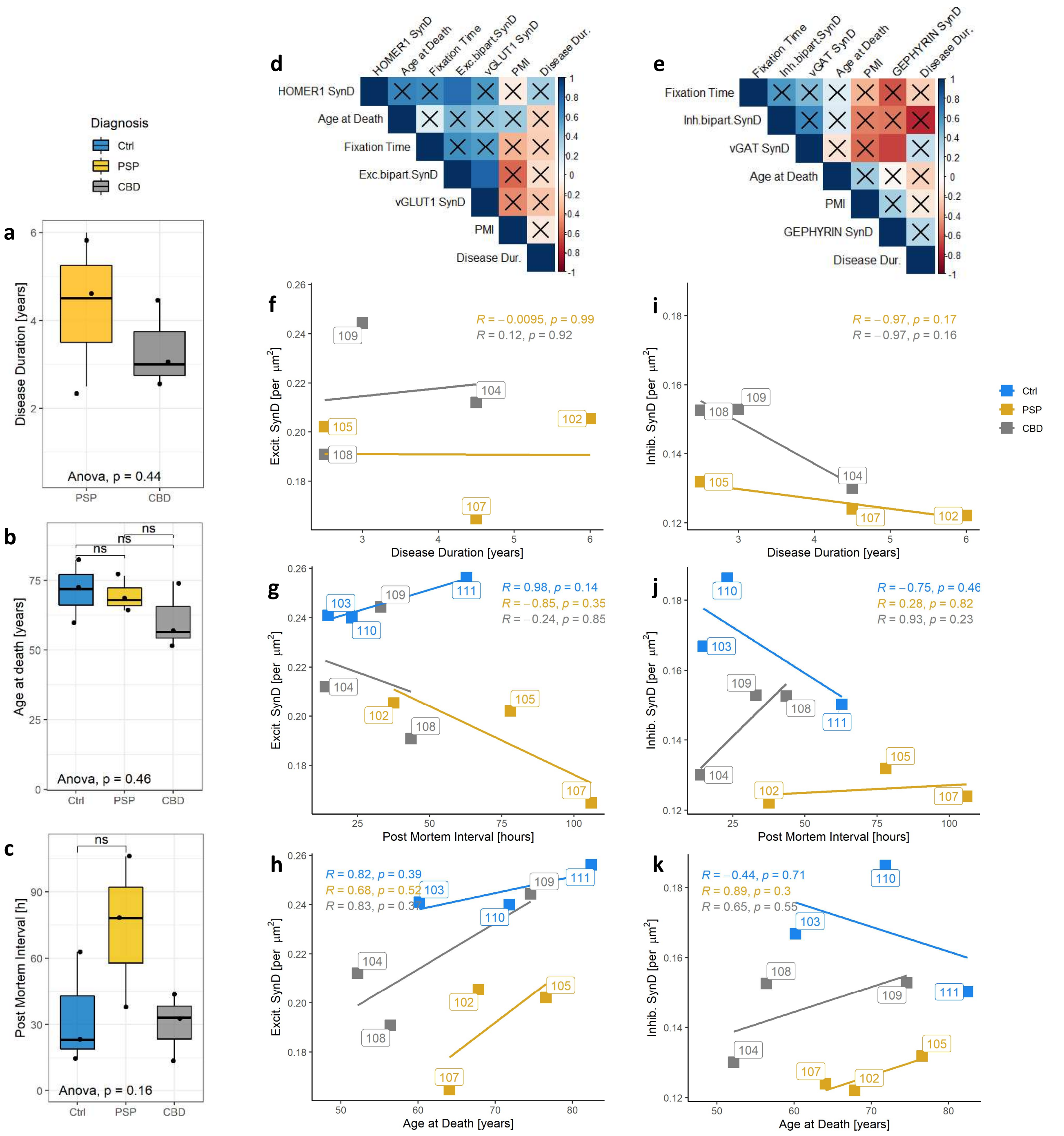

Suppl. figure. 1. Comparison of subject covariates.

(a)-(c) Pair-wise comparisons of control, PSP and CBD cohorts regarding disease duration **(a)**, age at death in years **(b)** and post mortem interval (PMI) **(c)** of samples used for synapse analysis show no significant differences between cohorts. The upper and lower hinges of each box correspond to the 75th and 25th percentiles, while median values are represented by the black bar. Whiskers display the range of data within 1.5 of the inter-quartile range, correspondent to the full range of distribution of all 3 cases in the cohort. One-way-ANOVA and Welch-test were used to evaluate group differences, where 'ns' > 0.05

(d)-(e) Correlation matrices indicate Pearson's R (shade) and p-value > 0.05 (crosses) for pairwise correlation tests between covariate data and synapse densities in an cohort-undifferentiated view for synapse counts in the fCtx. The order of features is given by hierarchical clustering results. Regarding the excitatory synapse analysis, only significant positive correlations between the density of bipartite synapses and pre- or postsynaptic densities are observed **(d)**. Regarding the inhibitory synapse analysis, no significant correlations but a negatively correlated pre- and postsynaptic density counts are apparent **(e)**.

(f)-(k) The densities of excitatory and inhibitory bipartite synapses in the fCtx are not significantly correlated with the covariates data in a cohort-differentiated view; neither with disease duration **(f,i)**, pmi **(g,j)** nor age at death **(h,k)**. Scatter plots of excitatory **(f)-(h)** or inhibitory synapse density **(g)-(i)** in the fCtx and given covariate. Color code indicates cohort assignment. Boxed labels show single case identifiers. Statistical results are expressed as Pearson's R and respective decimal p-values (see also table 1).

Abbreviations: bipart.- bipartite, Dur. – duration, Exc. – excitatory, Inh. – inhibitory, SynD – synapse density.

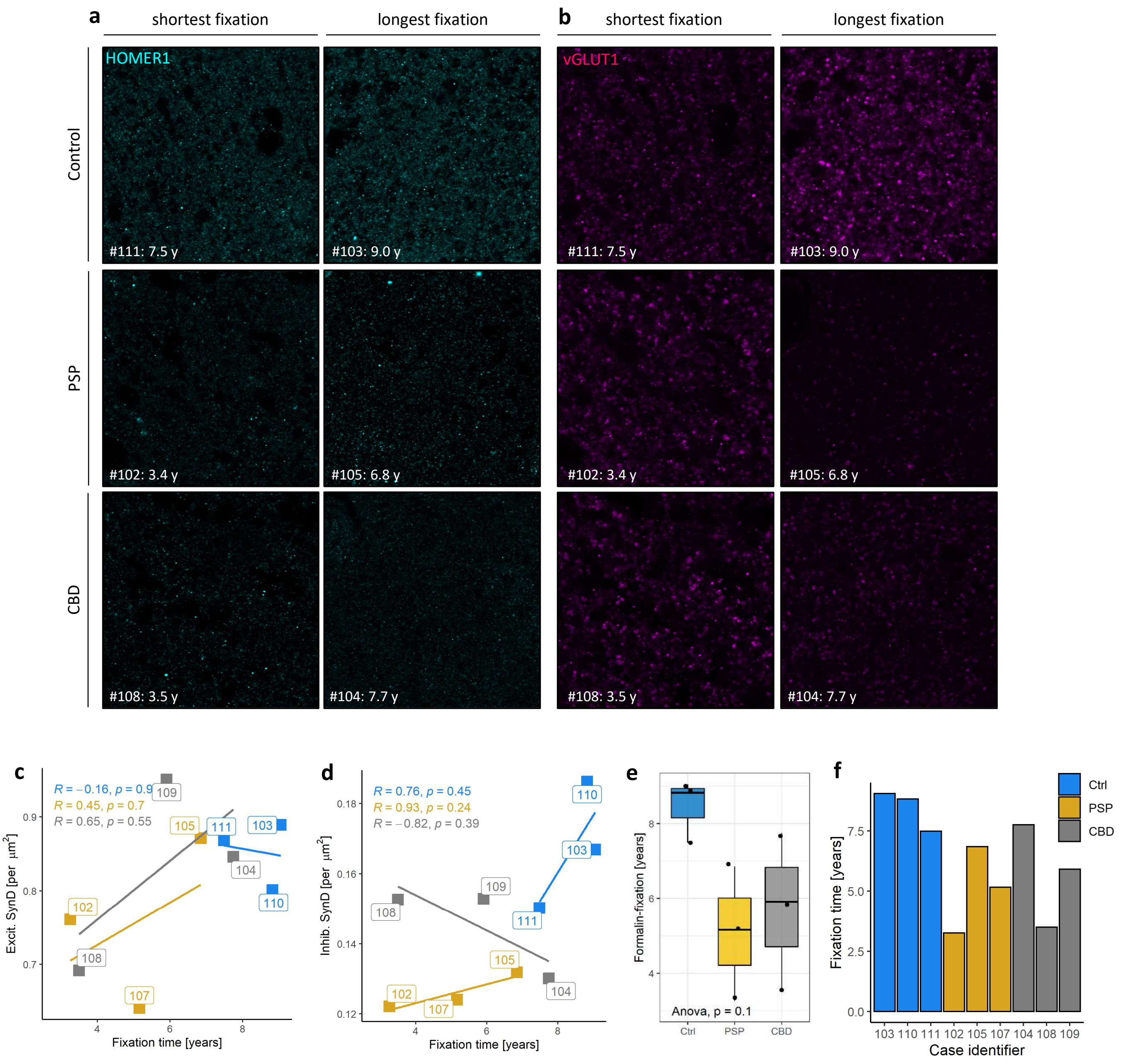

Suppl. figure 2. Influence of fixation time.

(a)-(b) Representative confocal images of the excitatory bipartite synapse immunofluorescent staining (HOMER1 **(a)**, vGLUT1 **(b)**) in the fCtx of control, PSP and CBD samples. For each excitatory synapse marker those samples are shown, which were fixed the shortest (respective left column) or the longest (respective right column) within the respective cohort given in rows. Text insets show case identifiers and fixation time in years [y].

(c)-(d) Scatter plots of the density of excitatory **(c)** or inhibitory **(d)** bipartite synapses in the fCtx and the time of formalin-fixation show no significant correlations. Color code indicates cohort assignment. Boxed labels show single case identifiers. Statistical results are expressed as Pearson's R and respective decimal p-values.

(e) Pair-wise comparisons of control, PSP and CBD cohorts regarding formalin-fixation time of samples used for synapse analysis show no significant differences between cohorts. The upper and lower hinges of each box correspond to the 75th and 25th percentiles, while median values are represented by the black bar. Whiskers display the range of data within 1.5 of the inter-quartile range, correspondent to the full range of distribution of all 3 cases in the cohort. One-way-ANOVA and Welch-test were used to evaluate group differences.

(f) Barplot of single case fixation times. The time in years ranges from 3.3 to 9 years.

Abbreviations: Exc. – excitatory, Inh. – inhibitory, SynD – synapse density.

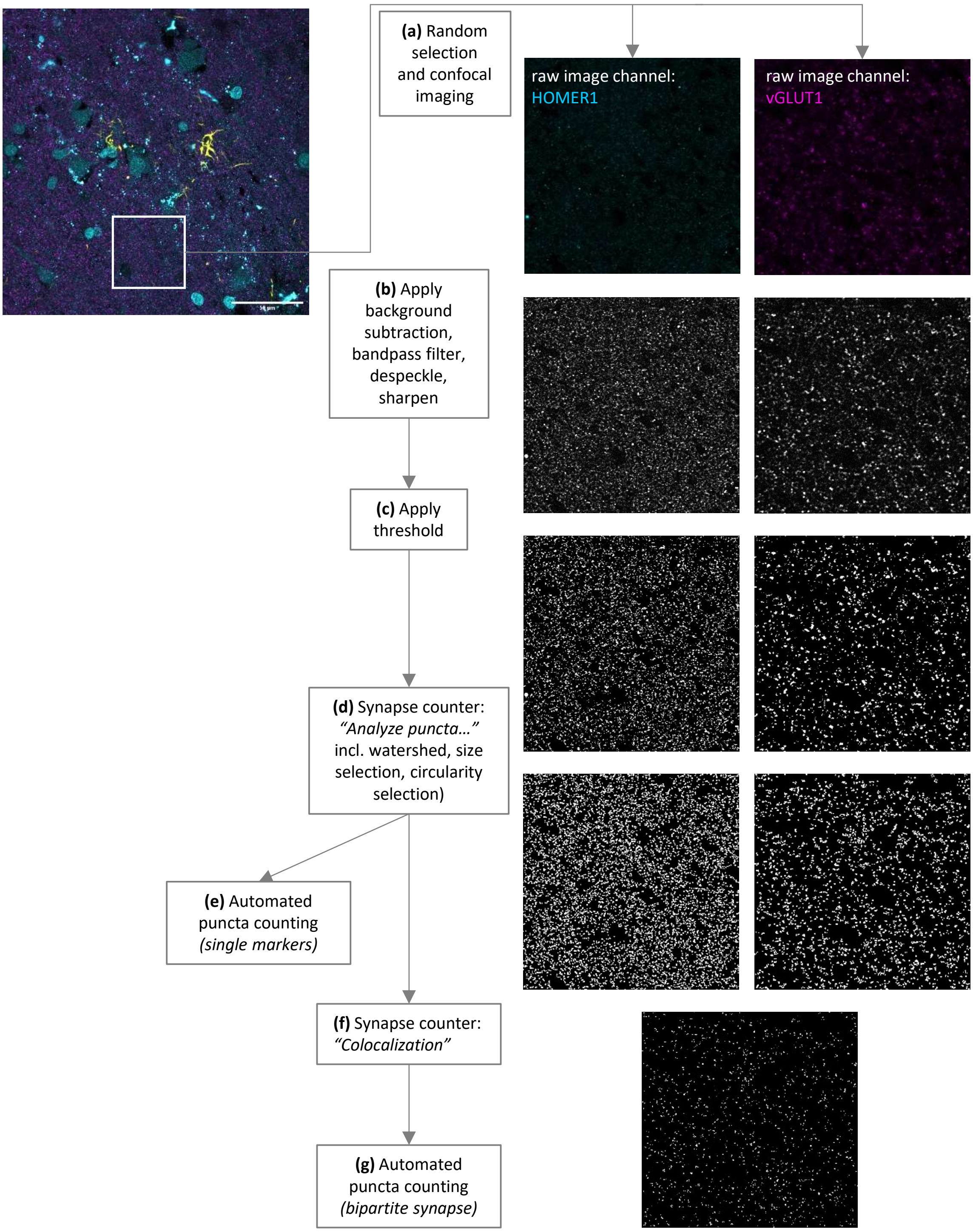

Suppl. figure 3. Image pre-processing and synapse quantification workflow.
The bipartite synapse densities were quantified from confocal imaging data followed by a consecutive digital image (pre-)processing workflow.
Starting with the light-sheet confocal imaging of a randomly selected area of 50 x 50 μm^2 **(a)** the raw image was preprocessed in Fiji/ImageJ by subtracting background noise, bandpass filtering, despeckling and sharpening **(b)**, as well as thresholding (Yen algorithm) for image binarization.
Then the *SynapseCounter* tool **(d)** was deployed to detect and count single puncta of synaptic markers **(e)** or to detect pre- and postsynaptic signal colocalization **(f)** in order to quantify corresponding bipartite synapses **(g)**.

CASE	Excit. Presynapses	Excit. Postsynapses	Excit. Bipartite Synapses	Inhib. Presynapses	Inhib. Postsynapses	Inhib. Bipartite Synapses
	**fCtx**					
Mean Ctrl	0.853	0.824	0.246	1.103	0.979	0.168
Mean PSP	0.758	0.727	0.191	0.980	1.084	0.126
P-value PSP: Ctrl	*0.288*	*0.099*	*0.038*	*0.395*	*0.155*	*0.048*
Mean CBD	0.829	0.695	0.216	0.963	1.093	0.145
P-value CBD:Ctrl	*0.791*	*0.072*	*0.186*	*0.340*	*0.111*	*0.161*
	**Str**					
Mean Ctrl	0.550	1.439	0.321	1.010	1.175	0.167
Mean PSP	0.532	1.308	0.282	1.123	1.125	0.147
P-value PSP:Ctrl	*0.689*	*0.099*	*0.284*	*0.022*	*0.533*	*0.095*
Mean CBD	0.395	1.338	0.209	1.123	1.167	0.145
P-value CBD:Ctrl	*0.201*	*0.310*	*0.183*	*0.650*	*0.923*	*0.267*

Supplementary table 1. Quantification of bipartite synapse density.
Mean values [per µm²] and p-values of excitatory and inhibitory synapses density analysis in the cortex of the middle frontal gyrus (fCtx, upper part) and in the striatum (Str, lower part) in PSP, CBD and control brains.
Abbreviations: Exc. – excitatory, Inh. – inhibitory.
